# Diurnal Encounter‐Based Size Distribution, Nesting Sites and Habitat Characteristics of the Mugger Crocodile ( *Crocodylus palustris* ) in Beeshazari Lake Complex, Nepal

**DOI:** 10.1002/ece3.71486

**Published:** 2025-06-10

**Authors:** Nikita Phuyal, Nishan KC, Bijaya Neupane, Bijaya Dhami, Mahamad Sayab Miya, Thakur Silwal, Gunjan Adhikari, Shraddha Pudasaini, Bishal Bhandari, Hari Adhikari

**Affiliations:** ^1^ School of Forestry and Natural Resource Management, Institute of Forestry Tribhuvan University Kathmandu Nepal; ^2^ WWF Nepal Kathmandu Nepal; ^3^ Institute of Forestry, Pokhara Campus, Tribhuvan University Pokhara Nepal; ^4^ Department of Forest Sciences, Faculty of Agriculture and Forestry University of Helsinki Helsinki Finland; ^5^ Department of Biological Sciences University of Alberta Edmonton Alberta Canada; ^6^ Department of Biological Sciences Western Kentucky University Bowling Green Kentucky USA; ^7^ College of Natural Resource Management Agriculture and Forestry University Udayapur Nepal; ^8^ Department of Geosciences and Geography University of Helsinki Helsinki Finland; ^9^ Forest Nepal Butwal Nepal

**Keywords:** basking, Beeshazari Lake complex, habitat characteristics, relative abundance index, submerged

## Abstract

Mugger crocodiles are the apex predator species of the wetland ecosystem in Nepal, and their conservation could safeguard the entire ecosystem. However, studies on their population status and habitat characteristics are limited, with no scientific research conducted on their nesting ecology to date. Therefore, we selected muggers as a representative species to better understand their daytime sightings, nesting characteristics, and the fine‐scale anthropogenic and environmental factors influencing their occurrence in five lakes of the Beeshazari Lake complex (BLC; Beeshazar Lake, Kumal Lake, Tikauli Lake, Kingfisher Lake, and Batuli Pokhari) of Chitwan National Park, Nepal. We conducted a preliminary survey, followed by a daytime sightings survey, a nesting site survey, and a habitat assessment survey in March 2023. A generalised linear model under binary logistic regression was used to analyse the factors influencing the habitat characteristics of muggers. During the research period, 50 detections of muggers were recorded, 66% of which were observed basking and 34% were submerged in the lakes. The Relative Abundance Index of the muggers in the BLC was 3.29 km^−1^. Nesting sites (two from Tikauli Lake and three from Beeshazar Lake) were recorded during the study period. The probability of sighting a mugger was significantly influenced by the slope (moderate slope), substrate type (clay, grass, and sand), mid‐lake depth, presence of anthropogenic threats, and presence of invasive species. We recommend that future researchers employ more robust models, such as N‐mixture models, to provide up‐to‐date information on the population abundance of muggers in the BLC. Furthermore, a comprehensive multi‐seasonal study focusing on ecological and behavioural aspects of nesting sites alongside environmental aspects influencing nest success rates is critical. Such research will be crucial in guiding the development of targeted conservation strategies aimed at protecting and preserving essential nesting sites. Moreover, we recommend conducting robust studies on the carrying capacity of wetlands in Nepal to provide insights into the sustainable population size that a wetland can support.

## Graphical abstract

This study assessed the diurnal encounter‐based size distribution, nesting sites, and habitat features of the mugger crocodiles in five lakes of the Beeshazari Lake complex. Key factors such as slope, substrate type, mid‐lake depth, anthropogenic threats, and invasive plant species significantly affected mugger occurrence, highlighting the need for targeted conservation strategies.

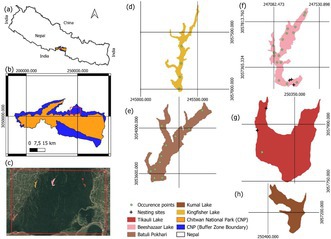

## 1 Introduction

Of the 24 species of extant crocodylians globally, Nepal is home to two species, namely the mugger crocodile ( *Crocodylus palustris* ) and the gharial ( *Gavialis gangeticus* ) (Nishan et al. 2023; Lamichhane et al. 2022). The mugger crocodile (hereafter mugger) occupies the slow‐flowing freshwater ecosystem with a wide range of habitat types, including rivers, lakes and marshes, reservoirs, irrigation canals, man‐made ponds, coastal saltwater lagoons, and estuaries (Whitaker 1987; Whitaker and Whitaker 1989). The mugger, often called the marsh crocodile, occurs in India, Nepal, Pakistan, and Iran, with a varying population status (Da Silva and Lenin 2010). They play a key role in the structure and function of freshwater ecosystems. They are essential for nutrient distribution across the waterbodies, which enhances primary production and supports healthy fish populations (Mulozoki 2000). They are listed as vulnerable under the IUCN Red List of Threatened Species and are listed in Appendix 1 of the Convention on International Trade in Endangered Species of Wild Fauna and Flora (CITES) (Choudhury and De Silva 2013). Muggers tend to bask in the midstream on rocks or muddy banks (Groombridge and Wright 1982). During winter, they usually spend their day basking on the rocky, sandy, and clay banks with their mouths open for both thermoregulation purposes and intra‐species communication (Price et al. 2022).

Muggers were relatively common in the past throughout the tropical lowlands (Terai ) of Nepal, where suitable habitat was protected and illegal hunting and anthropogenic disturbance were minimal (Shah and Tiwari 2004). However, due to severe anthropogenic threats and habitat modification, their current national population is restricted within the habitats of Chitwan National Park (CNP) in central Nepal, Bardia National Park (BNP), Shuklaphanta National Park (ShNP) and Ghodaghodi Lake Complex (GLC) in western Nepal, and the Koshi Tappu Wildlife Reserve (KTWR) in eastern Nepal (Bhattarai et al. 2022; Khadka et al. 2014; Lamichhane et al. 2022; Rawat et al. 2020). Some studies on mugger population estimation have been conducted by earlier researchers (Andrews and McEachern 1994; Maskey and Schleich 1992). The total mugger population was predicted to be between 400 and 500 individuals in Nepal (Baral and Shah 2013). However, according to the latest published information, 26 muggers were recorded in the GLC (Lamichhane et al. 2022); 397 muggers within and around CNP (Khadka and Lamichhane 2022) and 35 muggers in and around KTWR (Bhattarai et al. 2022). All these sub‐populations are at risk of declining due to ongoing conservation threats and challenges.

The primary threats to muggers include rapid agricultural and industrial development, habitat destruction, drowning in fishing nets, egg predation by local people, and the use of crocodile parts for medicinal purposes (Groombridge and Wright 1982). In Nepal, the reduction of wetland areas, deposition of silt and sediments, deterioration of water quality, eutrophication, and mortality in fisheries are key conservation challenges for crocodilian species, including muggers (Andrews and McEachern 1994; Nishan et al. 2023; Khadka et al. 2014; Shrestha 2000; Dhami, Maraseni et al. 2023). Despite the Beeshazari Lake complex (BLC), a Ramsar‐listed and UNESCO World Heritage Site, being one of the prime wetland habitats in CNP for globally threatened fauna, including muggers (Khadka and Lamichhane 2022; Rajbhandari and Acharya 2014), for the past few years, it has been increasingly disturbed by growing tourism activities. During our field visit, we found that the lake complex has been pressured by immense anthropogenic disturbances, including recreation activities (jungle walks, jeep safaris), illegal fishing, and dependency on forest products (fodder, fuelwood, and fiddlehead ferns). Moreover, the increased intensity of plastic pollution, siltation, eutrophication, fluctuating water level, and invasive alien plant species has deteriorated the quality of the lake ecosystem.

Few studies have been conducted on muggers regarding their distribution and habitat characteristics in Nepal. For example, Nishan et al. (2023) assessed the habitat selection and conservation threats of muggers in the Rapti River of CNP, while Bhattarai et al. (2022) studied their status, distribution, and habitat use in KTWR, and Lamichhane et al. (2022) examined their population status, habitat characteristics, and conservation threats in the GLC. It is necessary to regularly monitor the population status of muggers to assess species trends and patterns over the year. Similarly, information on age classes and behavioural activities is also equally important for developing strategies to help muggers adapt to changing environments, ensuring their survival in the long term. Moreover, information on mugger nesting ecology is crucial for understanding their habitat preferences and responses to anthropogenic factors (Choudhary et al. 2018; Vasudevan 1998), and understanding nesting characteristics is key to maintaining priority conservation areas. Information on nesting characteristics has implications for the conservation and monitoring of nesting sites during the reproductive season, which could improve the survival rates and contribute to the conservation of the species (Mazzotti et al. 2022). The BLC, consisting of interconnected lakes, swamps, and marshes, provides critical habitats for endangered bird species and water sources for large mammals. Additionally, the surrounding forest serves as an essential corridor and refuge, facilitating the movement of large mammals from CNP to the Siwalik region (Siwakoti and Karki 2010). Ecological features of the BLC differ significantly from those of previously studied sites, where past research primarily focused on population estimates (Khadka et al. 2014; Khadka and Lamichhane 2022) and habitat characteristics (Rajbhandari and Acharya 2014). Previous studies on mugger have been limited to a single lake. In contrast, our research encompasses five lakes within BLC. Thus, examining habitat characteristics in the BLC will provide valuable new insights regarding the important factors influencing mugger occurrence in one of the World Heritage Sites.

The BLC, a Ramsar site designated in 2003, has undergone substantial ecological restoration through the collaborative efforts of the Buffer Zone Management Committee and park authorities. This restoration has resulted in a significant increase in both domestic and international tourism, primarily focused on avitourism and wildlife safaris, contributing positively to local economies. However, this heightened anthropogenic pressure has also introduced challenges, including increased pollution, the introduction of invasive species, and general ecosystem disturbance. For example, studies have documented plastic ingestion by vulnerable megafauna, such as the one‐horned rhinoceros (*Rhinoceros unicornis*) (Awasthi et al. 2023), and the proliferation of invasive plant species, such as *Mikania micrantha* (Murphy et al. 2013). These anthropogenic impacts underscore the urgent need for evidence‐based management strategies (e.g., visitor management strategies) to ensure the long‐term ecological integrity and functionality of the BLC ecosystem.

The information regarding anthropogenic and environmental factors influencing the occurrence of threatened species is crucial to better inform timely habitat management (Dhami, Neupane et al. 2023; Nishan et al. 2023). Furthermore, understanding the factors that a species prefers or avoids can aid in targeted conservation efforts for apex predators such as muggers in both the immediate and the long term. As indicated by past studies, some factors that influence the habitat characteristics and distribution of muggers include lake width, lake depth, substrate type, water current, land topography, human disturbances, nesting sites, and invasive species (Bhattarai et al. 2022; Nishan et al. 2023; Lamichhane et al. 2022). However, there is very limited information on how these factors influence habitat characteristics and the distribution of apex predators, such as muggers, in multiple lake clusters. As apex predators, muggers play a critical role in regulating ecosystem dynamics by exerting top‐down control on prey populations and influencing trophic interactions. Their conservation is therefore essential for maintaining the ecological integrity and biodiversity of the BLC. A critical step toward effective conservation is developing a comprehensive understanding of the species' ecological requirements, particularly concerning habitat characteristics and nesting ecology. Thus, the primary aim of our study is to generate baseline information on the nesting characteristics of muggers within the selected study site. Furthermore, our study aims to generate comprehensive information on the habitat characteristics of muggers in the BLC by analysing the important anthropogenic and environmental factors that influence their occurrence. Such knowledge will aid park authorities and other conservation stakeholders in implementing effective wetland management strategies to support a viable mugger population and, in turn, contribute to the conservation of other threatened aquatic fauna that share this wetland ecosystem.

## 2 Materials and Methods

### 2.1 Study Area

The study was undertaken in the BLC of CNP. The CNP, declared a UNESCO World Heritage Site in 1994, is located in the southern part of central Nepal and currently covers an area of 953 km^2^. The BLC (27°37′04.6″ N, 84°26′11.3″ E) is a part of the Barandabhar biological corridor that connects CNP in the south and the Mahabharat Mountains to the Annapurna Himalayan region in the north (Lamichhane et al. 2013; Pandey 2012). The lake complex encompasses an area of 32 Km^2^, including the mosaics of diverse habitats of open waterbodies, marshes, swamps, grasslands, and Sal ( *Shorea robusta)* ) forests (Lamichhane et al. 2016). The Khageri River is the main natural drainage of the BLC. The vegetation of the study area has been categorised as Sal mixed forest, grasslands, and riverine forests (Government of Nepal 2014; Verheugt 1995). The study area has a subtropical climate with three major seasons: summer, monsoon, and winter. The area's average temperature is 34°C (Lamichhane et al. 2016). The mean annual rainfall is 2150 mm (Lamichhane et al. 2016).

**FIGURE 1:**
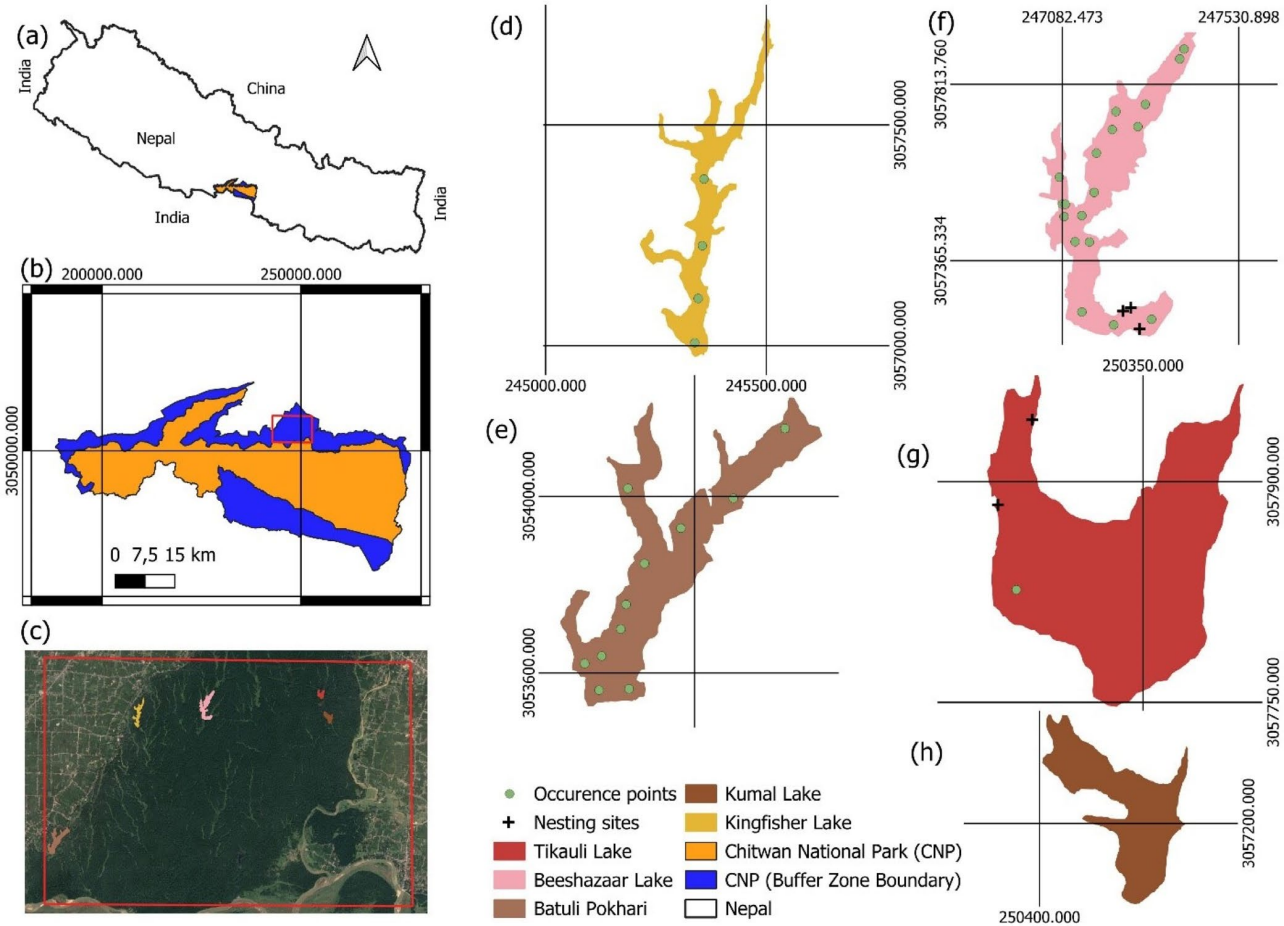
Study area map showing: (a) boundary of Nepal, (b) Chitwan National Park with its buffer zone, (c) Beeshazari Lake complex indicating the locations of sampled lakes, (d) Kingfisher Lake, (e) Batuli Pokhari, (f) Beeshazaar Lake, (g) Tikauli Lake, and (h) Kumal Lake. The dark plus signs indicate the nesting sites, and green circles denote the occurrence points of mugger crocodiles.

The lake complex has been recognised as a global biodiversity hotspot, supporting a pristine habitat for several globally threatened species, including the Bengal tiger ( *Panthera tigris tigris* ), one‐horned rhinoceros ( *R. unicornis* ), Asian elephant ( *Elephas maximus* ), mugger crocodile ( *C. palustris* ), and the critically endangered birds such as white‐rumped vulture ( *Gyps bengalensis* ) and lesser adjutant stork ( *Leptoptilos javanicus* ). The lake system provides irrigation water to nearly 60,000 farmers in western Chitwan. The major ethnic groups residing in the lake complex are the Bote, Majhi, and Darai (Acharya 2010; Nepal and Weber 1995). The current survey was conducted in Beeshazar Lake, Kumal Lake, Tikauli Lake, Kingfisher Lake, and Batuli Pokhari, which tend to serve as pristine habitats for mugger crocodiles, as shown in Figure 1.

### 2.2 Sampling Design and Data Collection

#### 2.2.1 Preliminary Survey

The preliminary field visit was conducted in March 2023 to familiarise researchers with the locations and routes of the study sites and to plan the major field surveys and measurements. Initially, all 20 lakes of the BLC were surveyed, and later, five lakes of the complex that tend to serve as prime habitats for muggers were chosen for a systematic survey. We conducted in‐depth informal interviews with four key CNP conservation officials and five National Trust for Nature Conservation (NTNC)–Biodiversity Conservation Center (BCC) officials. These interviews helped us to gain an overview of the current distribution of muggers within the lake complex. Each lake of the complex was visited during the preliminary field visit. During the visit, the GPS coordinates of each lake were stored as reference points. Later, with the help of these reference points, the lake boundaries were digitised using Google Earth Pro 7.3.3.

#### 2.2.2 Mugger Occurrence Survey

Based on information obtained from the preliminary survey, daytime sightings of muggers were conducted in five lakes of the complex (Beeshazar, Batuli Pokhari, Kumal, Tikauli, and Kingfisher). Of the total lakes in BLC, only those with sufficient water availability and those that tend to serve as prime habitats for mugger crocodiles were included in the systematic survey. The survey was carried out from 9:00 to 17:00 h during the daytime in the late winter season or early spring (March 2023) for 10 days to maximise the probability of sightings coinciding with the basking time of the mugger (Bhattarai et al. 2022; Nishan et al. 2023; Lamichhane et al. 2022; Neupane et al. 2020). A four‐person group of experts and research assistants surveyed each lake of the complex in turn in a sequential method during the daytime. Most of the lakes are separated by some land mass (> 500 m), along with anthropogenic infrastructures in between, which is why frequent movement of muggers from one lake to another was not expected throughout the survey period (DNPWC 2018). Moreover, we assumed that muggers would be less active during the winter months, so we did not expect them to move frequently during the field survey period (Lamichhane et al. 2022). This reduced the chances of duplicating individual crocodile recordings during the surveys. A traditional dugout canoe was used rather than engine boats to avoid disturbing the muggers and to maximise sighting probability. Three lakes (Kumal, Tikauli, and Kingfisher) accessible on foot were surveyed without a canoe. Nevertheless, a dugout canoe was used on the larger lakes (Beeshazar and Batuli Pokhari) to increase the probability of sighting individuals throughout the lake area. Both sides of the lake were systematically sampled to determine the occurrence of the species. The GPS locations of mugger crocodiles observed on the opposite bank were recorded only once that bank was reached. Similarly, the GPS locations of crocodiles floating on the lake were recorded from the nearest bank, assuming it was the occurrence point (Lamichhane et al. 2022). The total length (TL) of each sighted mugger was visually estimated to categorise them into age classes: hatchlings (< 30 cm); yearlings (30–50 cm); juveniles (50–125 cm); subadults (125–180 cm); and adults (> 180 cm) following Bhattarai et al. (2022) and Khadka et al. (2014). Binoculars (Vortex optics 8 × 42 and Olympus 10 × 50) were used for the observations to determine individual details (i.e., age class and activity–basking/submerged). Moreover, one research team member used a camera to capture the live activities (either basking or submerged) of the muggers; hence, photographic documentation was used to record the muggers' behavioural activities.

#### 2.2.3 Nesting Site Survey

The mugger is a hole‐nesting species that lays eggs during the annual dry season (typically mid‐March to early April) (Khadka et al. 2014). The nesting survey was therefore carried out in late winter or early spring (March 2023) for five days, covering five lakes of the complex. The nest locations were determined through a two‐phase approach. First, nests were located with the help of an experienced local tracker (Bhote). Bhote are a local indigenous community who largely depend on lakes for their livelihoods. In parallel, photographic evidence of the nests was captured. In the second phase, these photographs were cross‐verified by an experienced researcher with over 20 years of work with crocodilian species, who is affiliated with the Gharial Breeding Center at Kasara, Chitwan. This combined approach of local expertise and expert verification strengthens the reliability of the nest location data (DNPWC 2018).

Crocodile tracks and the presence of dug‐out soil on top were key indicators of potential nesting sites (Vasudevan 1998). The geographical location of each nest was recorded with the help of a Garmin GPS device. The details of the nesting site (substrate type, slope, aspect, distance from water sources, and distance from basking site) were also recorded. However, we could not record the nest parameters, such as nest depth and chamber width, to avoid the possible risk of a mugger attack. The substrate type of the sighted nesting sites was recorded based on visual estimation. Slope was measured using clinometers, and the aspect was measured using a compass. The distance from the nearest water sources and the distance from the basking site were measured using a range finder.

#### 2.2.4 Habitat Assessment Survey

**FIGURE 2:**
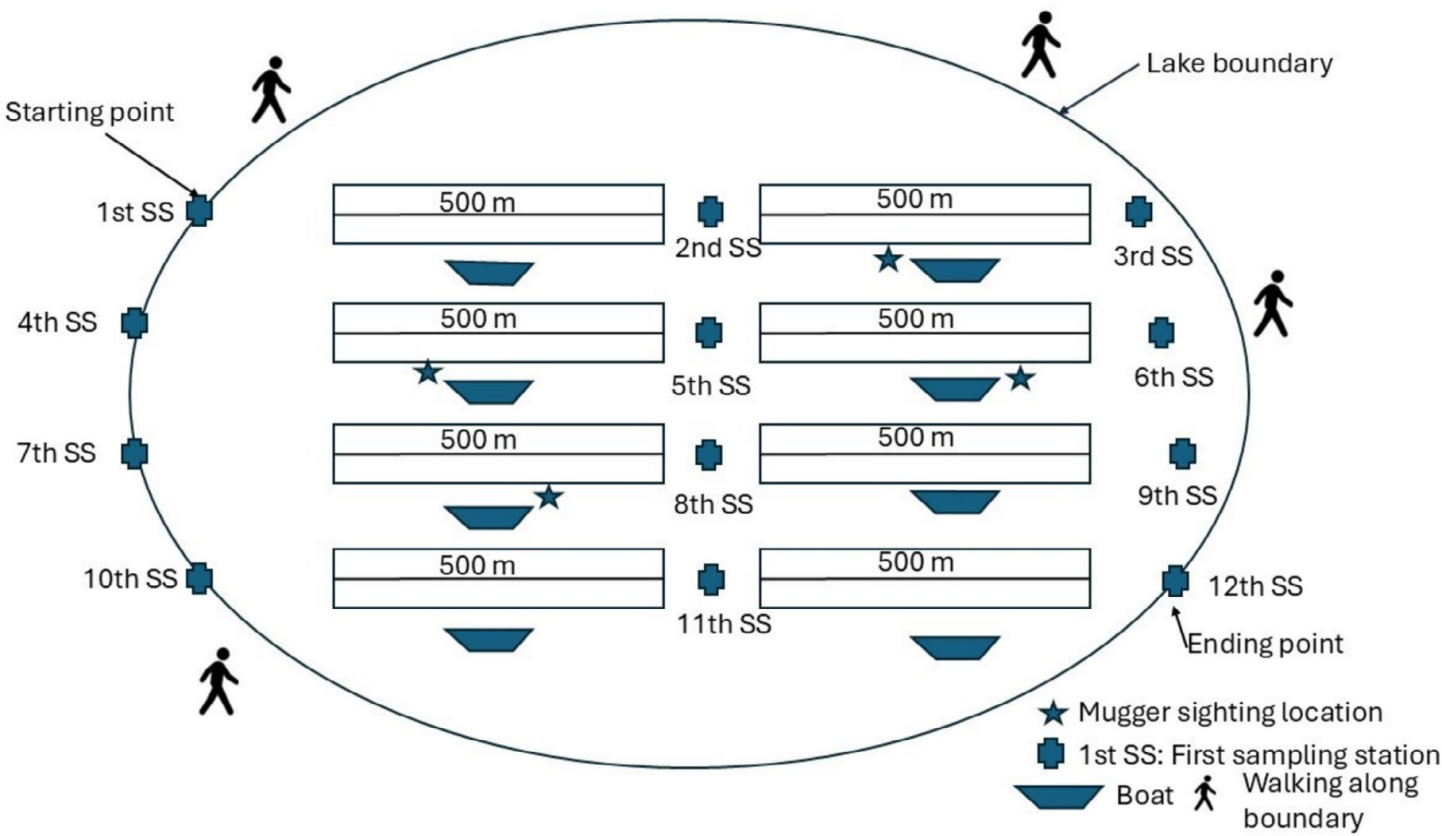
Diagram showing the layout of the horizontal transects and designed sampling stations for recording the habitat parameters of mugger crocodiles. The transects were surveyed from the boat. The first transect was chosen with a coin toss, and subsequent transects were conducted alternately.

**FIGURE 3:**
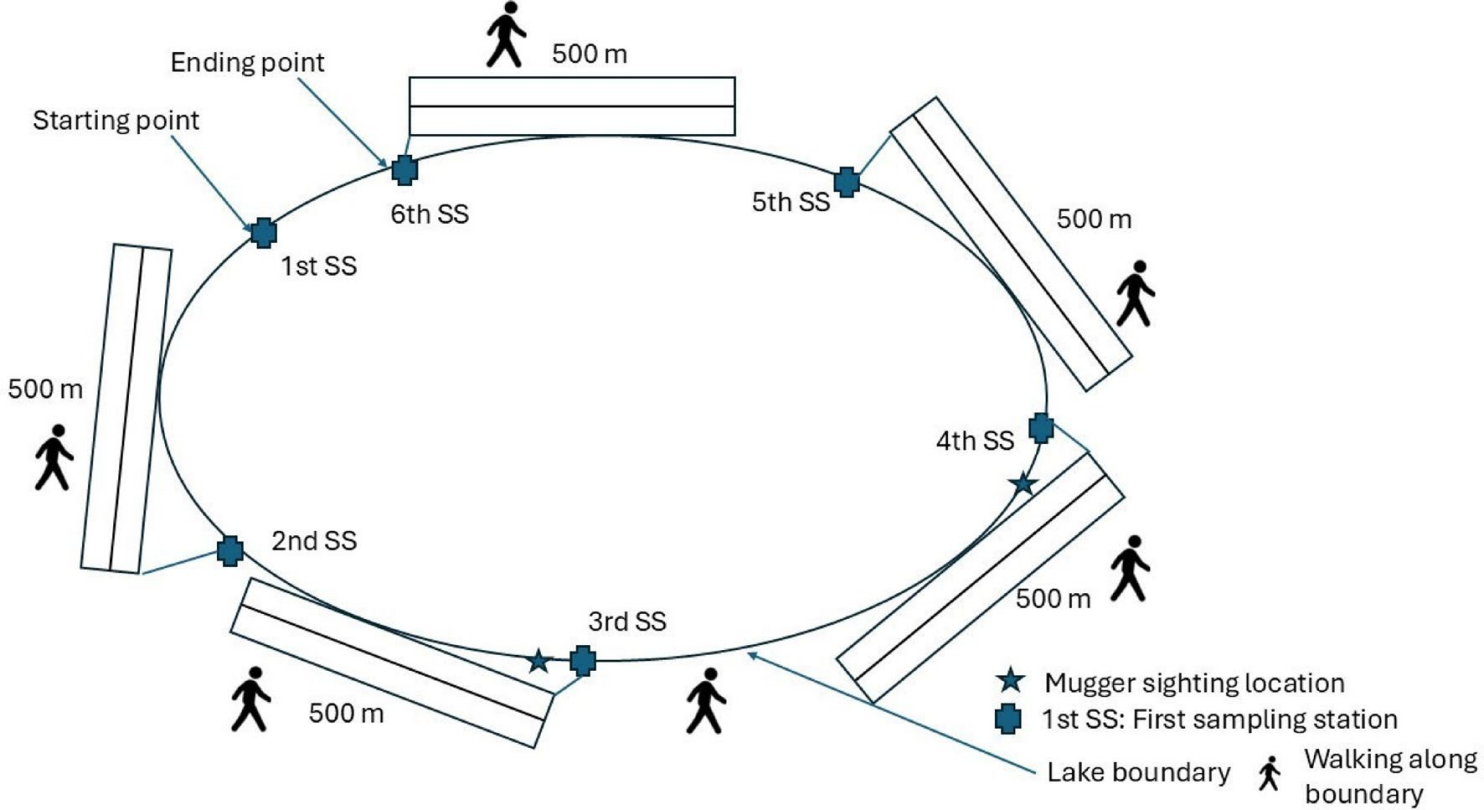
Diagram showing the layout of transects and sampling stations designed to record the habitat parameters of mugger crocodiles surveyed by walking. The first transect was chosen by a coin toss, and subsequent transects were conducted alternately.

For the detailed habitat characteristics recording, we generated sampling stations spaced at 500 m intervals along the boundary of three lakes (Kumal, Tikauli, and Kingfisher) using ArcGIS 10.8 (ESRI 2020) by assuming that the habitat characteristics would differ at an interval of 500 m. However, for the remaining lakes (Beeshazar and Batuli Pokhari), whose lake widths are greater than 1000 m, we first designed horizontal transects, and sampling stations were generated every 500 m along the transect. Habitat characteristics were noted even from the water using a traditional dugout canoe (Figure 2). However, due to the inaccessibility of the dugout canoe, habitat characteristics were recorded at each designated sampling station and at locations where muggers were sighted by walking along the boundary lines of the three surveyed lakes (Tikauli, Kumal, and Kingfisher) (Figure 3). We assumed that the probability of basking individuals moving across the spaced consecutive stations during the survey was small. Similar methods and assumptions have been adopted by multiple studies for mugger and gharial crocodiles (Bhattarai et al. 2022; Nishan et al. 2023; Lamichhane et al. 2022; Neupane et al. 2020).

Habitat characteristics were carefully recorded on both sides of the lake at predetermined sampling stations spaced at 500 m intervals and at each mugger sighting location. The survey was conducted simultaneously with the mugger occurrence survey. At each sampling station and sighting location, seasoned field guides with prior experience in researching crocodilian species were asked to scan each station for at least two minutes using binoculars and cameras.

Based on the preliminary survey and intensive literature review (Bhattarai et al. 2022; Nishan et al. 2023; Lamichhane et al. 2022; Neupane et al. 2020), nine pre‐defined habitat characteristics were recognised as potentially influencing mugger occurrences: slope, substrate type, lake width, mid‐lake depth, anthropogenic disturbances, invasive species, aspect, availability of nesting sites, and proximity to human settlement (Table 1). GPS coordinates were recorded for each sampling station and sighting location. The slope on both sides of the lake was recorded, and averaged to calculate the final slope. It was then classified into five slope categories: gentle (< 15°), moderate (15°–25°), moderately steep (25°–35°), steep (35°–55°) and very steep (> 55°) [adopted from Nishan et al. (2023)]. Similarly, the observed lakeside substrate was classified into nine categories: sandy bank (fine sand); sandy and grassy bank (fine sand sparsely covered with *Sachharum* spp. and other shrubs); gravel bank (loose aggregation of small stones); sand and gravel bank (fine sand with sparse gravel); clay and sandy bank (mixture of fine clay and sand); grass and forest bank ( *S. robusta* and riverine forest sparsely covered with *Sachharum* spp. and other shrubs); clay and grassy bank (fine clay sparsely covered with *Sachharum* spp. and other shrubs); grassy bank (covered with *Sachharum* spp. and other shrubs); gravel and grassy bank (gravel sparsely covered with *Sachharum* spp. and other shrubs) [adopted from Nishan et al. (2023)]. Lake width was measured using a range finder. Mid‐lake depth was measured using a 3 m long, graduated bamboo pole with measurements taken at 5 cm intervals as it was lowered to the lakebed, and the values were averaged for each sampling station. Additionally, we recorded the presence/absence of anthropogenic threats (illegal fishing, tourism and recreational activities, disposal of garbage and solid waste, and plastic pollution) and the presence/absence of invasive plant species occurring at each sampling station and sighting location (Bhattarai et al. 2022). At each sampling and sighting point, we marked a 1 m^2^ quadrat and measured the proportion of solid plastics and invasive plants. When their proportions were greater than the 30% threshold, we viewed it as evidence of pollution and invasion by invasive plant species. Even though we did not directly measure the chemical leaching of the disposed wastes, previous research in the same national park previously established macroplastics in faecal samples of various wildlife species, including one‐horned rhinoceros (Awasthi et al. 2023), thus confirming the general presence of plastic pollution in the environment. Moreover, the high‐resolution photographs taken of the observed invasive plants were identified by reviewing the relevant literature (Invasions of Alien Plant Species in Nepal) and verified (Shrestha and Shrestha 2021). The aspect was measured using a compass. The presence/absence of nesting sites located within the sampling stations and at mugger sighting locations was closely examined during the field survey. As for settlements, we extracted shapefiles from OCHA Nepal (https://bit.ly/3T9OuSC) and calculated the settlement distance from each sampling station and sighting location using the Euclidean distance tool in ArcGIS 10.8 (ESRI 2020).

**TABLE 1:** Detailed information on dependent and predictor variables used in the analysis. The presence and absence of muggers are dependent variables, and the rest are independent variables.

Variable	Variable category	Values	Data source
Mugger occurrence	Categorical	Detected = 1 Undetected = 0	Field survey
Slope	Categorical	Gentle (< 15°) Moderate (15°–25°) Moderately steep (25°–35°) Steep (35°–55°) Very steep (55°)	Field survey
Substrate type	Categorical	Sandy bank, sandy and grassy bank, gravel bank, sand and gravel bank, clay and sandy bank, grass and forest bank, clay and grassy bank, grassy bank, gravel and grassy bank	Field survey
Lake width	Continuous	Range (3–199.2)	Field survey
Mid‐lake depth	Continuous	Range (1.5–24)	Field survey
Anthropogenic threats	Categorical	Presence = 1 Absence = 0	Field survey
Invasive species	Categorical	Presence = 1 Absence = 0	Field survey
Aspect	Categorical	North Northeast East Southeast South Southwest West Northwest	Field survey
Availability of nesting site	Categorical	Presence = 1 Absence = 0	Field survey
Proximity to human settlement	Categorical	< 200 m 200–400 m 400–600 m 600–800 m	OCHA Nepal

### 2.3 Data Analysis

Since our study was based on a single detection survey using diurnal encounter‐based methods, it does not reflect the true population abundance of mugger crocodiles. Therefore, we used the Relative Abundance Index (RAI) as a proxy to standardise detection rates across survey sites. The RAI was calculated by dividing the total number of mugger detections by the total survey effort (in kilometres) (O'Brien et al. 2003).

Generalised linear models (GLMs) were used to examine the probability of mugger occurrence in relation to different habitat predictors at the study site. Mugger occurrence at sampling stations and sighting locations was the dependent variable. Similarly, the nine measured habitat variables (slope, substrate type, lake width, mid‐lake depth, anthropogenic threats, invasive species, aspect, availability of nesting site, and proximity to human settlement) were included as independent variables (predictors). Before the regression analysis, the multi‐collinearity test was computed for all the independent variables using the variance inflation factor (VIF) in the R package ‘Faraway’ (Boomsma 2014). As the VIF values were lower than 10, all the habitat variables were included in the final analysis (Bowerman and O'Connell 1990), see Table 2. The analysis considered all possible subsets of potential predictors (Barton and Barton 2015) that were ranked using the Corrected Akaike Information Criterion (AICc) of small sample size (Burnham and Anderson 2002).

**TABLE 2:** Variance inflation factor (VIF) values for habitat variables used for modelling.

Habitat variables	VIF
Lake width	1.167
Mid‐lake depth	1.871
Substrate type	1.649
Slope	1.206
Aspect	1.142
Availability of nesting site	1.515
Anthropogenic threats	1.478
Invasive species	1.263
Proximity to human settlement	1.259

We executed logistic regression analysis containing all the independent variables under the binomial family with the logit link function. We used the R package “MuMIn” to build models, and the best‐fitting model was identified by comparing the AIC value, with the lowest AIC value indicating the best fit (Kenny 2020). Top candidates' models with ΔAIC ≤ 2 were averaged to generate the final model‐averaged coefficient, containing the estimate, std. error, *Z* value, and Pr(>(|*z*|)) value for each independent variable. The independent variables exhibit a statistically significant association with the dependent variable if the *p*‐value (Pr(>|*z*|)) is less than 0.05 (at a 5% significance level). The predictive ability of the best‐fit model was assessed based on the area under the curve (AUC) of the receiver operating characteristic value range (1–0) by using the R package “ROCR” (Sing et al. 2005). Values that tend to lie between 0.7 and 0.8 were considered acceptable discrimination, 0.8–0.9 as excellent discrimination, and > 0.9 as superior discrimination (Hosmer and Lemeshow 2000). The R statistical software version 2023.06.2 (R Core Team 2023) was used to perform the modelling analyses.

## 3 Results

### 3.1 The Relative Abundance Index (RAI) of the Mugger Crocodile

The RAI of the mugger crocodile in the BLC was 3.29 km^−1^. Muggers were recorded from four lakes—Beeshazar, Tikauli, Kingfisher, and Batuli Pokhari—with RAIs of 8.76, 1.12, 1.34, and 3.96 km^−1^, respectively. Out of 50 detections, 33 were of muggers basking on the banks of the lakes, and six of these basking detections displayed mouth‐gaping behaviour. The remaining 17 detections were of muggers submerged in the lakes. The details of the mugger sightings are shown in Table 3.

**TABLE 3:** Details of mugger sightings in the Beeshazari Lake complex.

S. No.	Name of lake	Total mugger sighted	RAI	Age class	Activity of the mugger sighted in the lake
1	Tikauli Lake	1	1.12	1 (subadults)	1 (basking)
2	Beeshazar Lake	31	8.76	8 (subadults), 1 (juveniles), 22 (adults)	11 (submerged), 17 (basking), 3 (basking with gaping mouth)
3	Kingfisher Lake	4	1.34	4 (subadults)	2 (submerged), 1 (basking), 1 (basking with gaping mouth)
4	Batuli Pokhari	14	3.96	1 (juveniles), 13 (adults)	4 (submerged), 8 (basking), 2 (basking with gaping mouth)

### 3.2 Observed Number of Nesting Sites and Their Characteristics

**FIGURE 4:**
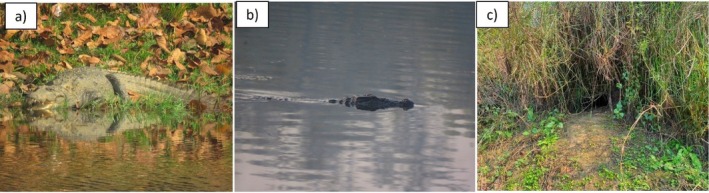
Mugger observed in the Beeshazari Lake complex; (a) Basking at Beeshazar Lake (15/03/2023), (b) Submerged at Batuli Pokhari (10/03/2023), (c) Nesting site spotted at Beeshazar Lake.

During the nest survey, we recorded a total of five nesting sites from two lakes: Tikauli (2 sites) and Beeshazar (3 sites) (Table 4). Of these, two nests were found in areas composed of clay, grass, and sand, while the other three were located in areas with just clay and grass. In terms of terrain, two sites were on gentle slopes, two on moderate slopes, and one on a steep slope. The nesting sites were located 20–35 m from water sources. Regarding the orientation (aspect) of the sites, one site each faced northwest, west, east, southeast, and north. Additionally, the nests were found 10–20 m away from basking sites (Figure 4).

**TABLE 4:** Detailed information of nesting sites in the Beeshazari Lake complex.

Nesting site	Location of nesting site	Dominant vegetation	Substrate type	Slope	Distance from water sources (m)	Aspect	Distance from basking site (m)
Nesting site 1	Tikauli Lake	*S. robusta* , *Terminalia elliptica*	Clay, grass, and sand	Gentle	30	Northwest	15
Nesting site 2	Tikauli Lake	*S. robusta* , *T. elliptica*	Clay, grass, and sand	Gentle	20	West	10
Nesting site 3	Beeshazar Lake	*S. robusta* , *T. elliptica*	Clay and grass	Moderate	25	East	20
Nesting site 4	Beeshazar Lake	*S. robusta* , *T. elliptica*	Clay and grass	Moderate	35	Southeast	20
Nesting site 5	Beeshazar Lake	*S. robusta* , *T. elliptica*	Clay and grass	Steep	30	North	20

### 3.3 Habitat Characteristics Associated With Observed Muggers

The observed number of mugger individuals according to the different habitat features is summarized in Appendices 2 and 3. Regarding lake width, the largest number of mugger individuals (*N* = 25; 50%) was recorded in the category of 50.01–100 m, whereas for mid‐lake depth, the most individuals (*N* = 22; 44%) was recorded at the depth range of 20–25 m. Similarly, clay and grassy banks were the substrate types where the most mugger individuals were found basking (*N* = 32; 64%). Regarding slope, we observed the largest number of muggers (*N* = 28; 56%) on gentle slopes. For the aspect category, muggers (*N* = 14; 28%) were most observed facing the south aspect. Most mugger individuals were seen on lake banks that were substantially less affected by anthropogenic disturbances and invasive alien plant species. Regarding nest site proximity, most mugger individuals were observed on lake banks proximal to nesting sites.

### 3.4 Key Habitat Factors Influencing Mugger Occurrence

Only two component models were developed through model averaging (models with ΔAIC ≤ 2), and the best‐fitted model (AICc = 86.59, Akaike model weight = 0.69) included mid‐lake depth, anthropogenic threats, invasive species, nesting site, proximity to human settlement, slope, and substrate type. Similarly, its competing model (AICc = 88.18, Akaike model weight = 0.31) included mid‐lake depth, anthropogenic threats, invasive species, nesting site, proximity to human settlement, slope, and substrate type. The outcomes of the model‐averaged coefficients (Table 5) showed that, out of the nine predictors, only slope (with a moderate slope), substrate type (clay, grass, and sand), mid‐lake depth, presence of anthropogenic threats, and presence of invasive species were significant predictors influencing mugger occurrence.

**TABLE 5:** Model‐averaged coefficients describing mugger occurrence.

Predictor	Estimate	Standard error (SE)	Adjusted SE	*Z* value	*p*
Intercept	1.702e+00	1.732e+00	1.752e+00	0.972	0.331253
Slope (moderate)	−2.446e+00	1.038e+00	1.051e+00	2.328	0.0199*
Slope (moderately steep)	−1.051e+00	7.846e−01	7.937e−01	1.324	0.1854
Slope (steep)	−1.923e+01	2.207e+03	2.233e+03	0.009	0.9931
Substrate type (clay, grass, and sand)	−2.549e+00	8.321e−01	8.418e−01	3.028	0.0024**
Mid‐lake depth	3.792e−01	1.058e−01	1.071e−01	3.542	0.00039***
Anthropogenic threats (present)	−2.752e+00	7.764e−01	7.855e−01	3.503	0.00046***
Invasive species (present)	−1.829e+00	7.217e−01	7.301e−01	2.505	0.0122*
Nesting sites (present)	−3.378e+00	1.811e+00	1.832e+00	1.843	0.0653
Proximity to human settlement	8.733e−04	9.663e−04	9.777e−04	0.8931	0.3717

*Note:* The significant values for variables influencing mugger occurrence (pr(>|*Z*| < 0.05)) are in bold. Signif. codes: 0 ‘***’ 0.001 ‘**’ 0.01 ‘*’ 0.05.

The area under ROC function (AUC) (Appendix 4) values for the full model (GLM with binary logistic regression) were estimated to be 0.96, with an accuracy value of 0.92, and were thus considered to be a superior test (Appendix 4).

## 4 Discussion

### 4.1 The Abundance of Mugger Crocodile

The CNP hosts the largest population of mugger crocodiles in Nepal, with a total count of 397 individuals spread across the Rapti and Narayani Rivers, the BLC, and various stagnant ponds within the buffer zone (Khadka and Lamichhane 2022). Previous research by Rajbhandari and Acharya (2014) documented 36 individuals specifically in Beeshazar Lake of the BLC. However, our recent study revealed 31 independent detections in that lake. This observed decline in population size may be linked to the increasing plastic pollution and the proliferation of invasive species, as indicated by the findings of our research. As the overall objective of our study was not the precise estimation of mugger crocodile population, the number of muggers recorded in our study was derived from a one‐time survey. The one‐time survey has been extensively followed by multiple studies in the context of Nepal for monitoring the population of gharial and mugger crocodiles (Bhattarai et al. 2022; Lamichhane et al. 2022; Neupane et al. 2020; Nishan et al. 2023). Population data derived from the one‐time survey are valuable for understanding their current distributions, thereby aiding in the development of conservation strategies and management interventions. However, for more accurate population abundance estimates, we recommend that future researchers employ more robust methodology, such as N‐mixture models, as these are known to yield more reliable abundance estimates of threatened species compared with traditional direct counting (Paudel et al. 2015).

Our study revealed a predominance of adult muggers, followed by subadults and juveniles, consistent with previous research in CNP (Nishan et al. 2023; Khadka et al. 2014). However, this contrasts with the findings of Rajbhandari and Acharya (2014), who reported more subadults. This discrepancy can be attributed to physical competition for basking spots, where larger, dominant crocodiles outcompete smaller ones, securing prime locations (Magnuson et al. 1979; Venugopal and Deviprasad 2003). This competitive behaviour likely influences the age structure observed during our study, highlighting the importance of basking sites in shaping mugger population dynamics. The strong presence of adults indicates a healthy reproductive base, which is crucial for long‐term population sustainability. Meanwhile, the lower proportions of subadults and juveniles may indicate slower growth and turnover rates (Dhami, Neupane et al. 2023).

The maximum number of individuals was reported during basking (66%), followed by being submerged in the lakes (34%), and 12% of the basking mugger individuals displayed mouth‐gaping behaviour. Our present observation is similar to previous studies in the Rapti River (Nishan et al. 2023), KTWR (Bhattarai et al. 2022; Goit and Basnet 2011), and the GLC (Lamichhane et al. 2022). As ectothermic organisms, crocodiles rely on external heat sources to regulate their body temperature (Seebacher et al. 1999). Basking allows them to absorb solar radiation, increasing their core body temperature and enhancing physiological functions like digestion and muscle activity. By opening their mouths wide, crocodiles increase the surface area exposed to the environment, facilitating evaporative cooling to prevent overheating, particularly during warmer periods (Spotila et al. 1977). However, the specific function of mouth‐gaping behaviour may vary depending on environmental factors and social interactions (Kofron 1993). The reliance on specific basking sites also highlights the importance of habitat conservation, as changes to these areas could directly impact crocodile health and population stability. Hence, conservation efforts should focus on protecting and managing critical basking habitats, ensuring they are sufficient and undisturbed.

### 4.2 Observed Number of Nesting Sites and Their Characteristics

We reported five nesting sites in total from Tikauli and Beeshazar Lakes. The nests were observed on clay, grassy, and sandy surfaces. Nesting site selection is crucial for the survival of the species. Muggers show hole‐nesting behaviour, laying eggs during the dry season (Whitaker and Whitaker 1989). Nests can be in a wide variety of habitats. Choudhary et al. (2018) also observed that muggers prefer clay and sandy substrates for nesting in the Katarniaghat Wildlife Sanctuary (KWS), India. Out of 59 mugger nests in India, 37% were noted from gravel/sand and 34% from a sand substrate (Whitaker and Whitaker 1989). Likewise, nests were observed on the sandy substrate of the Cauvery River in Melagiris, India (Gour et al. 2022). Sandy substrates are easier to dig and have deeper nests (Vashistha et al. 2021). The reason for choosing clay along with a sandy substrate for nesting in the BLC may be the natural clay and sandy soil composition of the area, and such a mixed substrate may also allow muggers to crawl more easily into the nest. Besides, sand has a lower moisture content and moderates extreme hot or cold environmental conditions. Furthermore, nesting in the sandy substrate will help maintain the incubation temperature of eggs, which affects egg development and consequently influences hatchling recruitment and the population structure (While et al. 2018). Our study is limited by the small sample size of nesting sites, which may not fully capture the broader nesting behaviours of muggers. Further research with a larger sample size and extended duration is needed to gain a comprehensive understanding of nest success rates, predation pressures, and key nesting habitat characteristics. Nonetheless, safeguarding and maintaining diverse nesting habitats, particularly clay and sandy substrates, is crucial for supporting successful reproduction and ensuring the long‐term viability of mugger crocodile populations.

Nests were more frequent on gentle (< 15°) and moderate (15°–25°) slopes. This may be related to the detection of muggers, as 56% were reported from gentle slopes and 32% were reported from moderate slopes. Nesting on gentle slopes may provide better temperature regulation for the eggs. Moreover, a gentle slope may allow for efficient drainage and prevent waterlogging, which could affect nest temperature. Muggers were found to preferably nest at a 12.8° ± 0.2° slope in KGS (Choudhary et al. 2018). Nest sites were found mainly facing east and west. Like in our study, Whitaker and Whitaker (1989) observed that nine out of eleven nests were located on east‐facing slopes in Amaravathi, India. Facing the east and west aspects may expose the nests to optimal sunlight for temperature control during various times of the day. This exposure can help regulate nest temperature and promote proper embryonic development. However, further research is necessary to investigate the possible reasons.

The nests were located 20–35 m from water sources and 10–20 m from basking sites. Similar to our results, Choudhary et al. (2018) reported that muggers prefer to nest 20.37 ± 0.075 m from the water in KWS, India. In another study in Iran, mugger nests were located a few metres from the water's edge (Mobaraki et al. 2013). Patil et al. (2012) documented a nest of muggers at 3.04 m from the water in the Kadavi River in India. In contrast to our findings, Vasudevan (1998) found nesting sites located 10–250 m in 1994 and 10–650 m in 1976 from the water level, in Amaravathi, India. Whitaker and Whitaker (1984) reported that nests were located at an average of 10 m from the shore (range 1 m–2 km) in Tamil Nadu, India. In a captive study in Pakistan, muggers dug the nest chamber quite far from the waterbody (Qazi and Barkati 2014). The potential reason for nesting far from water may be an adaptation against flooding or water level rise, while nesting far from basking sites may be to prevent nests from being damaged by other adults. However, further research is needed to validate the reason for nesting site selection at a certain distance from basking sites and waterbodies.

Our study provides baseline information on nesting sites in the BLC, which will allow future researchers to conduct more comprehensive studies on nesting ecology. However, nesting site selection depends on multiple factors, including ecological aspects, such as prey availability and predator presence, and behavioural aspects, such as courtship displays and mate selection. So, to address these limitations, future studies should adopt a holistic approach by integrating ecological and behavioral data alongside environmental parameters to safeguard critical mugger nesting sites in the BLC, which will eventually support the long‐term survival of the mugger crocodile in its natural habitat (Bashyal et al. 2024; Khadka et al. 2020). We acknowledge that both ecological and behavioural processes, like female experience, body condition, and site fidelity, also shape nest choice (Combrink 2014; Seymour and Ackerman 1980). Previous studies indicate that experienced females may recapitulate successful locations, while first‐time nesters may select differently. Because we concentrated on habitat characteristics, we did not incorporate behavioural observations or individual nesting histories. Subsequent research needs to include these elements with extended monitoring to comprehensively explain mugger nesting ecology.

### 4.3 Key Habitat Factors Influencing Mugger Occurrence

We examined the influence of nine predictors associated with the probability of mugger occurrence at the BLC to understand the species' habitat characteristics to devise effective conservation strategies for muggers.

According to our modelling analysis, lake bank slope was one of the important predictors for mugger occurrence, as a moderately inclined slope (15°–25°) was found to have a statistically negative influence on the occurrence probability. In contrast to our study, Nishan et al. (2023) reported that the mugger occurrence probability significantly increased on the banks of the Rapti River in CNP with moderate slopes (15°–25°). This could be attributed to the difference in terrain and substrate features between the banks of the Rapti River and the BLC. Banks of the BLC are largely occupied by gently inclined terrain with a clay and grass substrate, which could make it easier for muggers to crawl to the lake. Bhattarai et al. (2022) and Lamichhane et al. (2022) reported the insignificant influence of bank slope on mugger occurrence at KTWR and GLC, respectively. The clay, grassy, and sandy banks have a negative influence on the probability of mugger occurrence in our study area. During our study, we only recorded two substrate types where muggers were observed: clay and grassy banks, and clay, grassy and sandy banks. The majority (64%) of the basking muggers were observed at clay and grassy banks. Muggers prefer to bask on a clay and grassy substrate, as such combinations may provide them with an optimum temperature during basking. However, muggers are reported to bask on a variety of substrate types, including fallen logs, wet mud, sand, and gravel banks (Khadka et al. 2014). According to our modelling analysis, mid‐lake depth significantly positively influenced the occurrence of muggers in the study area. A similar result has also been observed by Bhattarai et al. (2022) at KTWR. The lake bank with a higher water depth can often be safer for muggers, allowing them to bask for longer periods. This could be attributed to the fact that whenever basking muggers detect nearby threats, they can quickly dive into deep water. Similar findings were also supported by Hussain (2009) for gharials. Moreover, lakes with deeper water depths can provide suitable microhabitats for larger fish assemblages than those with shallower water depths (Miranda 2011). During our study period, we observed 44% of the muggers at a lake depth ranging from 20 to 25 m and 32% at a lake depth ranging from 15 to 25 m. The presence of nearby anthropogenic threats also significantly influences the occurrence of muggers in our study area. Crocodilians are cryptic, secretive, and easily disturbed by human presence (Read et al. 2007). During our study period, muggers avoided the lake banks where tourism activities, including jeep safaris, fishing, and plastic pollution, were prevalent (as per the field observations). Therefore, we suggest that park and buffer zone authorities should promote responsible nature tourism by adopting more eco‐friendly strategies, including reusable and biodegradable products, sustainable waste management, and wildlife‐friendly jungle safari tours and walks to minimise anthropogenic disturbances in the study area. The findings derived from our study will help wildlife managers and policymakers implement site‐based action plans and management strategies. However, we recommend that future researchers investigate the carrying capacity of the wetlands to provide insights into the sustainable population size that the local wetland can support.

The presence of invasive species also significantly influences the occurrence of muggers in our study area. The abundant invasive alien plant species of BLC included *Eichhornia crassipes* , sedge (*Cyperus* sp.), and water cabbage (*Pistia* spp.), which were primarily observed floating in the lakes. Such species can affect fish assemblages, one of the main prey species of muggers, by producing detritus and sediment capture (Chisholm and Moulin 2003; Gallardo et al. 2016).

## 5 Conclusion and Conservation Implications

Despite the limited sample size of nesting sites, our results provide valuable insights into key nesting habitat requirements, warranting further research to fully understand broader nesting behaviours and factors influencing nest success rates and nest predation pressures. Our analysis of habitat predictors revealed that factors such as slope (moderate), substrate type (clay and grassy), mid‐lake depth, presence of anthropogenic threats, and presence of invasive species significantly influence mugger occurrence. Moderate slopes with clay and grassy substrates provide optimal basking conditions, while deeper water depths offer protection from threats and suitable microhabitats for prey species. The negative impact of anthropogenic disturbances, such as tourism and plastic pollution, alongside the presence of invasive species, highlights the need for more sustainable tourism practices and effective management of invasive species in the BLC. Our study further recommends robust research relevant to the carrying capacity of wetlands to provide insights into the sustainable population size that a wetland can support.

## Author Contributions

**Nikita Phuyal:** conceptualisation, research design, data collection, data analysis and interpretation, drafting of the manuscript, critical review and revisions at different stages. **Nishan KC:** data analysis and interpretation lead, drafting of the manuscript, critical review, and revisions at different stages. **Bijaya Neupane:** conceptualisation, methodology design, manuscript drafting lead, and revisions at different stages. **Bijaya Dhami:** drafting of the manuscript, critical review, and revision at different stages. **Mahamad Sayab Miya:** drafting of the manuscript, critical review, and revision at different stages. **Thakur Silwal:** drafting of the manuscript, critical review, and revision at different stages. **Gunjan Adhikari:** drafting of the manuscript, critical review, and revision at different stages. **Shraddha Pudasaini:** drafting of the manuscript, critical review, and revision at different stages. **Bishal Bhandari:** drafting of the manuscript, critical review, and revision at different stages. **Hari Adhikari:** methodology design, GIS mapping lead, review draft, and edit.

## Conflicts of Interest

The authors declare no conflicts of interest.

## Data Availability

Data are available on https://github.com/adhihari/Data_NikitaArticle.

## Appendix 1 Lake Area, Perimeter Length, and the Respective Number of Stations

**Table:**

S. No.	Lake name	Lake perimeter (m)	Area of lake (m^2^)	No. of stations
1	Kumal Lake	1230.16	27042.49	2
2	Tikauli Lake	891.53	19674.25	2
3	Beeshazar Lake	3536.99	87513.91	7
4	Kingfisher Lake	2977.35	37016.48	5
5	Batuli Pokhari	3531.68	101048.32	7

## Appendix 2 Number of Sampling Stations, Sighting Locations, and Sighting Points During the Detailed Habitat Survey

**Table:**

Description	Number
Sampling stations	23
Sampling points at sampling stations	46
Mugger sighting locations between the stations	34
Sampling points at mugger sighting locations (both lake sides)	68
Total number of sampling points to record the habitat characteristics	114

## Appendix 3 Habitat Characteristics and Number of Muggers Observed in the Beeshazari Lake Complex

**Table:**

Habitat characteristics	Number of observed individuals (*N*)		Percentage (%)
Lake width (LW)			
< 50 m	1	25	2
50.01–100 m			50
100.01–150 m	23		46
150.01–200 m	1		2
Mid‐lake depth (MLD)			
< 5 m	1		2
5–10 m	2		4
10–15 m	9		18
15–20 m	16		32
20–25 m	22		44
Substrate type			
Clay grassy bank	32		64
Clay, grassy, and sandy bank	18		36
Slope			
Gentle	28		56
Moderate	16		32
Moderately steep	5		10
Steep	1		2
Aspect			
East	1		2
North	0		0
Northeast	10		20
Northwest	6		12
South	14		28
Southeast	5		10
Southwest	6		12
West	8		16
Invasive alien plant species (IAPs)			
Present	14		28
Absent	36		72
Anthropogenic disturbances (AD)			
Present	8		16
Absent	42		84
Nesting sites (NS)			
Present	44		88
Absent	6		12
Distance to human settlement			
< 200 m	0		0
200–400 m	4		8
400–600 m	18		36
600–800 m	23		46
> 800 m	5		10
